# Analysis of Sensory Recovery of Neurotized Free Flaps in Cases of Head-Neck Reconstruction: Our Experience in a Tertiary Care Hospital

**DOI:** 10.7759/cureus.77584

**Published:** 2025-01-17

**Authors:** Aparajita Saha, Jiten K Mishra, Shamendra A Sahu, Moumita De, Abi Sindhuja, Nikita Birua

**Affiliations:** 1 Burns and Plastic Surgery, All India Institute of Medical Sciences, Raipur, Raipur, IND

**Keywords:** gan, labc, neurotisation, neurotised free flap, sensory recovery in head and neck reconstruction

## Abstract

Functional outcomes have been better concerning swallowing ability, distinguishing hot and cold sensations, articulation, and improved range of tongue movement by sensory neurotization of the flaps used for head and neck reconstruction. We evaluated the sensory recovery in free flaps utilizing the great auricular nerve as the recipient sensory nerve, anastomosing a few fascicles of the greater auricular nerve (GAN) to that of the flap sensory nerve; lateral antebrachial cutaneous nerve in the radial forearm free flap, and lateral femoral cutaneous nerve in the anterolateral thigh flap. The sensory examination was performed at the visible center of the flap at the three-month and six-month follow-ups. The flaps were assessed for the recovery of five sensory modalities: fine touch, pain, pressure, two-point discrimination (2PD), and temperature. The sensory recovery was evaluated according to the Mackinnon Dellon scale. The sensory recovery in the neurotized flaps was compared with that in non-neurotized free flaps at the six-month follow-up. The sensory recovery graded by the Mackinon Dellon scale for all five modalities of sensation was statistically significant in sensate-free flaps at the six-month follow-up. Also, the sensory territory of the greater auricular nerve at the peri-auricular area was assessed for loss of sensation at the six-month follow-up, which was not clinically significant. We observed the great auricular nerve as a potential sensory nerve that may be utilized at the flap reconstruction site to neurotize free flaps used for head and neck reconstruction with a significant sensory recovery and without worrisome symptoms at the GAN sensory territory.

## Introduction

The primary objective of head and neck reconstruction is to restore not only the anatomy but also the function and aesthetics of the affected area [1]. Free tissue transfer is the preferred choice, especially in cases of head and neck oncoresection where the defect is complex so local and regional options may not suffice [2]. Microvascular surgery enables precise tissue transfer and provides vascular tissue by vascular anastomosis near the high-flow vessels, facilitating improved wound healing and better patient recovery [1-3]. This shift toward free flaps has been driven by the need for higher levels of rehabilitation and improved outcomes for patients undergoing head and neck reconstruction, particularly in cases involving complex defects and tissue damage from radiotherapy or malignancies [3-5].

One promising avenue in head and neck reconstruction is the use of neurotized free flaps, which incorporate sensory nerves of the flap anastomosed to the sensory nerve at the recipient site, which involves procedures like direct coaptation or with a nerve graft and thereby offer the potential for sensory recovery post-reconstruction [6,7]. This also depends on the availability of a recipient sensory nerve at the reconstruction site and donor sensory nerve, which is incorporated in the flap during flap harvesting.

By the neurotization of flaps used for reconstruction, outcomes have been better concerning the ability to distinguish hot and cold sensations, articulation, and improved range of tongue movement. The return of sensation within the oral cavity also leads to reduced chances of ulceration due to burns or bites [6].

In this clinical study, neurotization of free flaps is done to assess sensory recovery, that is, the nerve of the flap (donor nerve) is coapted with the greater auricular nerve (GAN) as the recipient nerve. Microneural anastomosis is done under microscope.

## Materials and methods

The study design was a comparative ambispective study, conducted after approval from the Institute Ethics Committee of AIIMS Raipur, on patients operated between March 2019 to March 2023. Thirty patients were included in our study. Fifteen patients were included in the study group in whom the neurotization procedure was done and the rest 15 were included in the control group in whom neurotization was not done. The primary objective of this study is to analyze the sensory recovery in neurotized free flaps in head and neck reconstruction in the follow-up period.

The secondary objectives were to compare the sensory recovery in neurotized free flaps with that of non-neurotized free flaps and to assess the sensory territory of the greater auricular nerve at the side where it was used as the flap recipient nerve.

All patients with age greater than 18 years of age with head-neck tumor reconstruction with a neurotized free flap (free radial artery forearm flap and free antero-lateral thigh flap) were included in the study. Exclusion criteria are patients who are lost to follow-up, not consenting to the procedure, patients with flap loss, patients where the flap is placed deep inside the oral cavity and inaccessible for sensory assessment, and patients who expired in post-op or within three months post-op. Patient demographic details were noted like age, sex, location of tumor, and type of tumor.

Neurotization procedure

All patients underwent reconstruction by the same surgical team. While raising the free radial artery forearm flap, the lateral antebrachial cutaneous nerve, which supplies the flap territory was harvested and the lateral femoral cutaneous nerve was incorporated with the free anterolateral thigh flap. A few fascicles of the greater auricular nerve were used for end-to-end coaptation with the sensory nerve of the flap. The greater auricular nerve fascicles (2-3) were coapted with the lateral antebrachial cutaneous nerve in case of the free radial artery forearm flap and lateral femoral cutaneous nerve of the thigh in anterolateral thigh free flap with nylon 10-0 suture under microscope magnification ranging 4.5x-8x with epineural sutures. Tension-free coaptation was done without the use of nerve grafts in all cases. Flap monitoring was done at regular intervals as per our department protocol, one hour on day 1, the second hour on day 2, and the third hour on day 3. There were no flap perfusion-related problems in any patients in this study, no re-exploration was done.

Sensory evaluation

All patients underwent radiotherapy following surgery. Patients were followed up at three-month and six-month intervals for sensory recovery over the flap. No patients were left to follow up in both groups. A sensory examination was performed at the visible and accessible center of the flap. In follow-up, sensory recovery was assessed by an independent observer who was unaware of the procedure, each time the same person assessed the patients. Patients were assessed based on five sensory parameters - touch, pain, pressure, two-point discrimination (2PD), and temperature. All parameters were used to evaluate final sensory recovery according to the Mackinon Dellon sensory scale (Table 1). Patients were asked to acknowledge sensation by holding up their fingers, and if no sensation was felt, to hold down their fingers depending on the sensation tested. Fine touch was evaluated by touching with a cotton swab. Pain was assessed by pricking with the sharpened edge of a toothpick. Temperature sensation was assessed with a cotton swab dipped in ice cold (5-10 degrees C) or warm water (45-50 degrees C). The temperature of the water (hot or cold) was checked with a cooking thermometer. Two-point discrimination was assessed with the sharpened ends of the divider. Distance between the ends was determined using a scale (starting with 20-30 mm). A pressure threshold test was done using standardized Semmes Weinstein monofilaments. Different monofilaments of variable thickness were used. Each filament was tested, starting with the lowest filament, tested at 90 degrees to the flap surface till the filament bends. The test was performed over the normal side first, then over the flap area with eyes closed. Filaments less than 4.08 were tapped more than thrice. Filaments of range 2.36-6.65 were used.

**Table 1:** The Mackinnon Dellon sensory scale used for all sensory parameters 2PD: two-point discrimination

Grade	Sensation	Static 2PD (mm)	Mobile 2PD (mm)
S_0_	No sensation in the territory of the nerve	-	-
S_1_	Perception of deep pain in the territory of nerve	-	-
S_1_^+^	Perception of superficial pain	-	-
S_2_	Perception of superficial pain and touch sensation	-	-
S_2_^+^	S2, with over sensation	-	-
S_3_	Perception of pain and touch with an absence of over-sensation	More than 15	More than 7
S_3_^+^	S3, with a perception of 2PD	7–15	4–7
S_4_	Complete	2–6	2–3

Statistical analysis

Data were analyzed using SPSS statistical software version 26.0 (IBM Corp., Armonk, NY, US). Categorical and continuous variables were expressed using frequency/percentage and mean/standard deviation (SD), respectively. The student's t-test, Mann-Whitney U test, and chi-square test (χ2) test were used. The level of statistical significance was set at a p-value of less than 0.05.

## Results

The mean age of participants in the study group was 47.13 years with an SD of 9.72 while the control group had a mean age of 46.87 years with an SD of 11.38. Both the study and control groups comprised 2 females (13.3%) and 13 males (86.7%). The location of the carcinoma and its type is given in Table 2. An anterolateral thigh (ALT) flap was done for 2 patients and a free radial artery forearm flap (FRAFF) for 28 patients. Sensory findings over the visible and accessible center of the flap at three months and six months follow-up have been elaborated in Table 3. P values were found to be significant for sensations of touch, pain, temperature, and pressure in the study group although two-point discrimination was found to be statistically insignificant between the two groups. The Mackinnon Dellon scale was also found to be statistically significant in the study group at six months. The GAN sensory territory was evaluated at six months postoperatively in patients in the study group. The results of sensory evaluation have been elaborated in Table 4. Although patients did not have any complaints over the GAN sensory territory, that is, it was clinically not significant, P value was found to be significant.

**Table 2:** Diagnoses and histopathology of patients in the study and control groups CA: cancer; SCC: squamous cell carcinoma; BCC: basal cell carcinoma

Diagnosis	Study			Control		
	N	%	Histopathology	N	%	Histopathology
CA left buccal mucosa	3	20	SCC	5	33.3	SCC
CA left preauricular area	1	6.7	BCC	0	0	0
CA lower lip with angle of mouth	2	13.3	SCC	0	0	0
CA over left forehead	0	0	0	1	6.7	BCC
CA right buccal mucosa	6	40	SCC	5	33.3	SCC
CA tongue	3	20	SCC	3	20	SCC
CA upper lip	0	0	SCC	1	6.7	SCC

**Table 3:** Results of the study and control groups showing all five sensory parameters, Mackinnon Dellon grading, and P value Sensory assessment of all patients with the Mackinnon Dellon scale with significant P values for all parameters 2PD: two-point discrimination

Sensory parameters		3 months (no. of patients)		6 months (no. of patients)		P value
		Present	Absent	Present	Absent	
Touch	Study	0	15	15	0	<0.01
	Control	0	15	5	10	
Pain	Study	0	15	15	0	<0.01
	Control	0	15	5	10	
Temperature-Hot	Study	0	15	15	0	<0.01
	Control	0	15	5	10	
Temperature-Cold	Study	0	15	15	0	<0.01
	Control	0	15	5	10	
Pressure	Study	0	15	15(mean-2.04g)	0	0.02
	Control	0	15	15(mean-226g)	0	
Static 2PD	Study	0	15	15(1.39cm)	0	0.75
	Control	0	15	15(1.6cm)	0	
Mobile 2PD	Study	0	15	15(0.72cm)	0	0.125
	Control	0	15	15(1.7cm)	0	
Mackinnon Dellon scale	Study	S0		S3(5),S3+(10)		<0.01
	Control	S0		S0(10),S2(4),S3(1)		

**Table 4:** Results showing sensory assessment of the GAN territory in the peripauricular area with the Mackinnon Dellon scale and P values Results of the sensory assessment of the periauricular area in the follow-up period showing no statistical significance 2PD: two-point discrimination

Sensory parameters	Present or Absent	GAN territory of the flap area	GAN territory of the contralateral area	P value
Touch	Present	15	15	1
	Absent	0	0	
Pain	Present	15	15	1
	Absent	0	0	
Temperature-Hot	Present	15	15	1
	Absent	0	0	
Temperature-Cold	Present	15	15	1
	Absent	0	0	
Pressure	Present	15(mean-42.56g)	15(mean-2.85g)	0.178
	Absent	15(mean-42.56g)	0	
Static 2PD	Present	15(mean-42.56g)	15(mean-0.55cm)	0.116
	Present	0	0	
Mobile 2PD	Present	15(mean-0.46cm)	15(mean-0.3cm)	1
	Absent	0	0	
Mackinnon Dellon Scale	Present	S3(1),S3+(4),S4(10)	S4(15)	0.049
	Absent	0	0	

**Figure 1:**
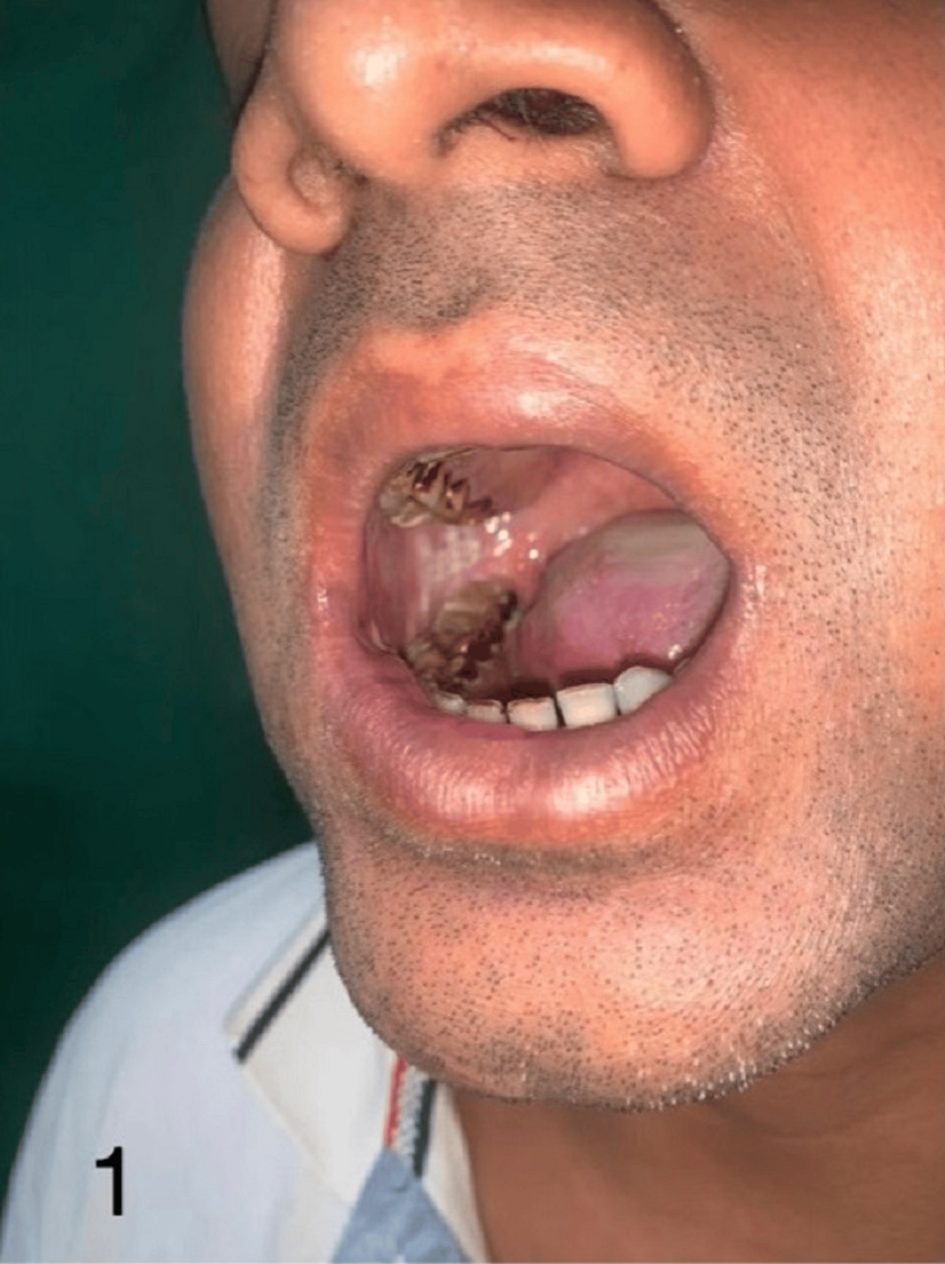
Preoperative image of a patient with SCC of the lower lip's right angle of the mouth planned for FRAFF SCC: squamous cell carcinoma; FRAFF: free radial artery forearm flap

**Figure 2:**
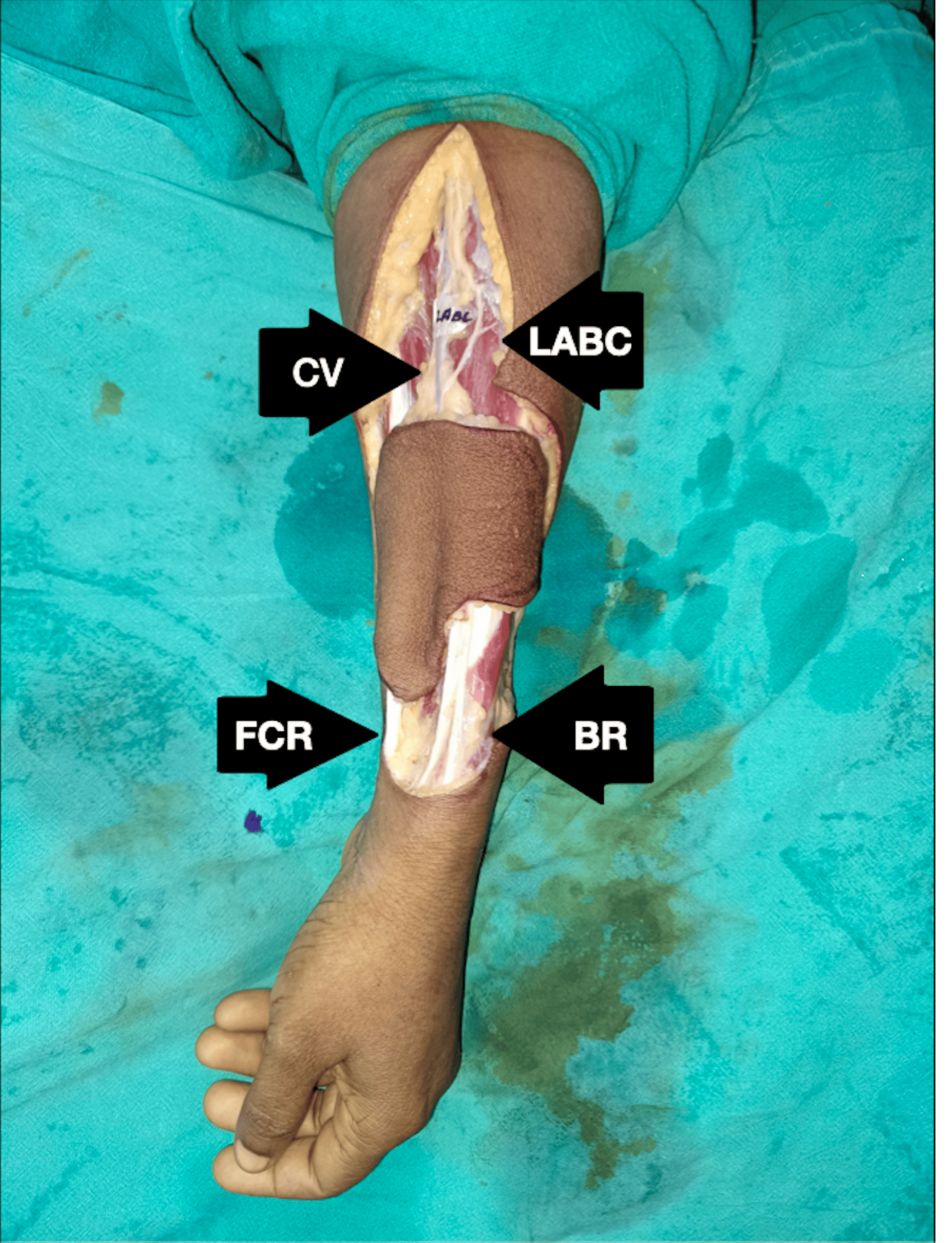
Intraoperative image showing flap harvest and relationship of the LABC nerve of the same patient LABC: lateral antebrachial cutaneous; FCR: flexor carpi radialis; BR: brachioradialis; CV: cephalic vein

**Figure 3:**
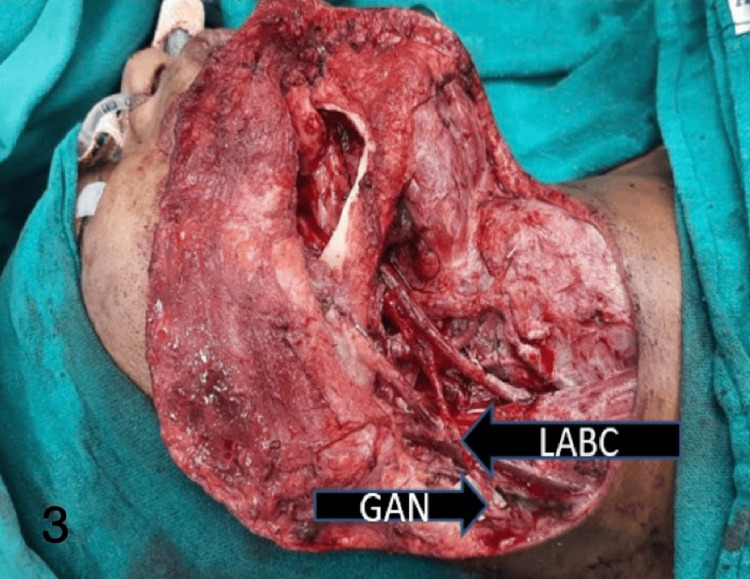
Intraoperative image showing end-to-end coaptation of LABC to GAN in a CA—cheek patient with marginal mandibulectomy and reconstruction with FRAFF LABC: lateral antebrachial cutaneous; GAN: greater auricular nerve; CA: cancer; FRAFF: free radial artery forearm flap

**Figure 4:**
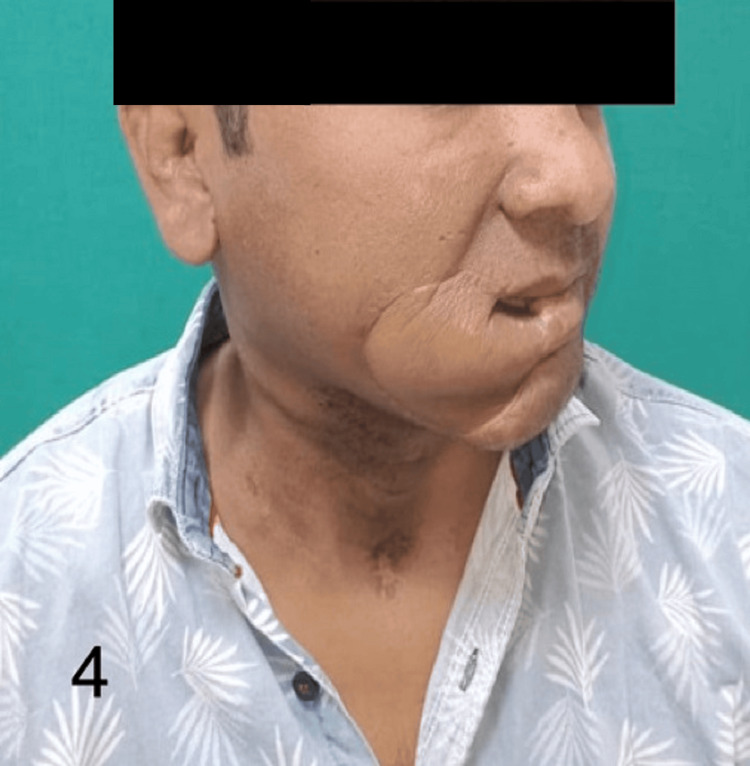
Follow-up image of the patient shown in Figure 1

Figures 1-4 show the preoperative, intraoperative, and postoperative images of a patient with SCC of the lower lip's right angle of the mouth.

**Figure 5:**
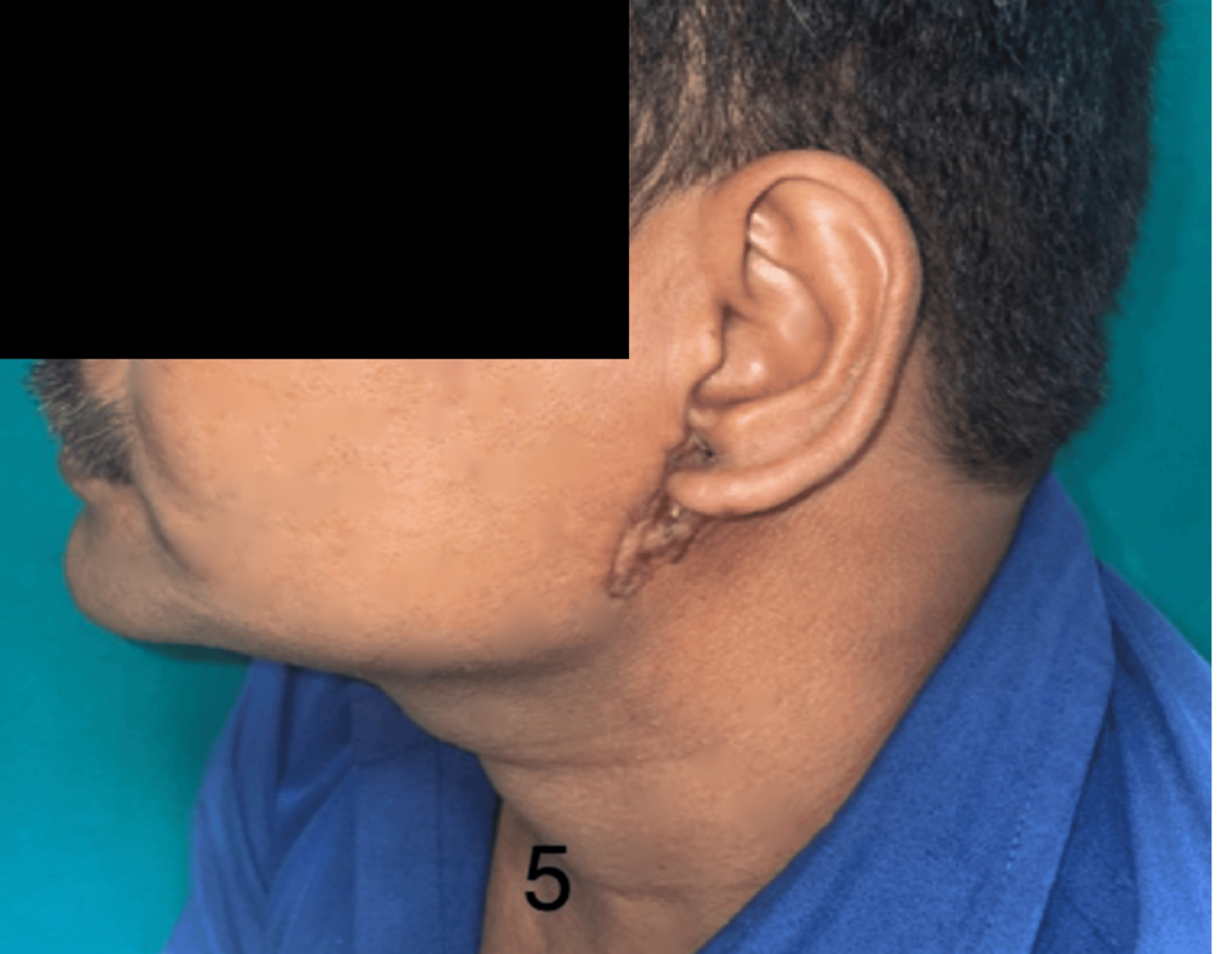
Preoperative image of a patient with BCC over the preauricular and retrooauricular area planned for free ALT flap BCC: basal cell carcinoma; ALT: anterolateral thigh

**Figure 6:**
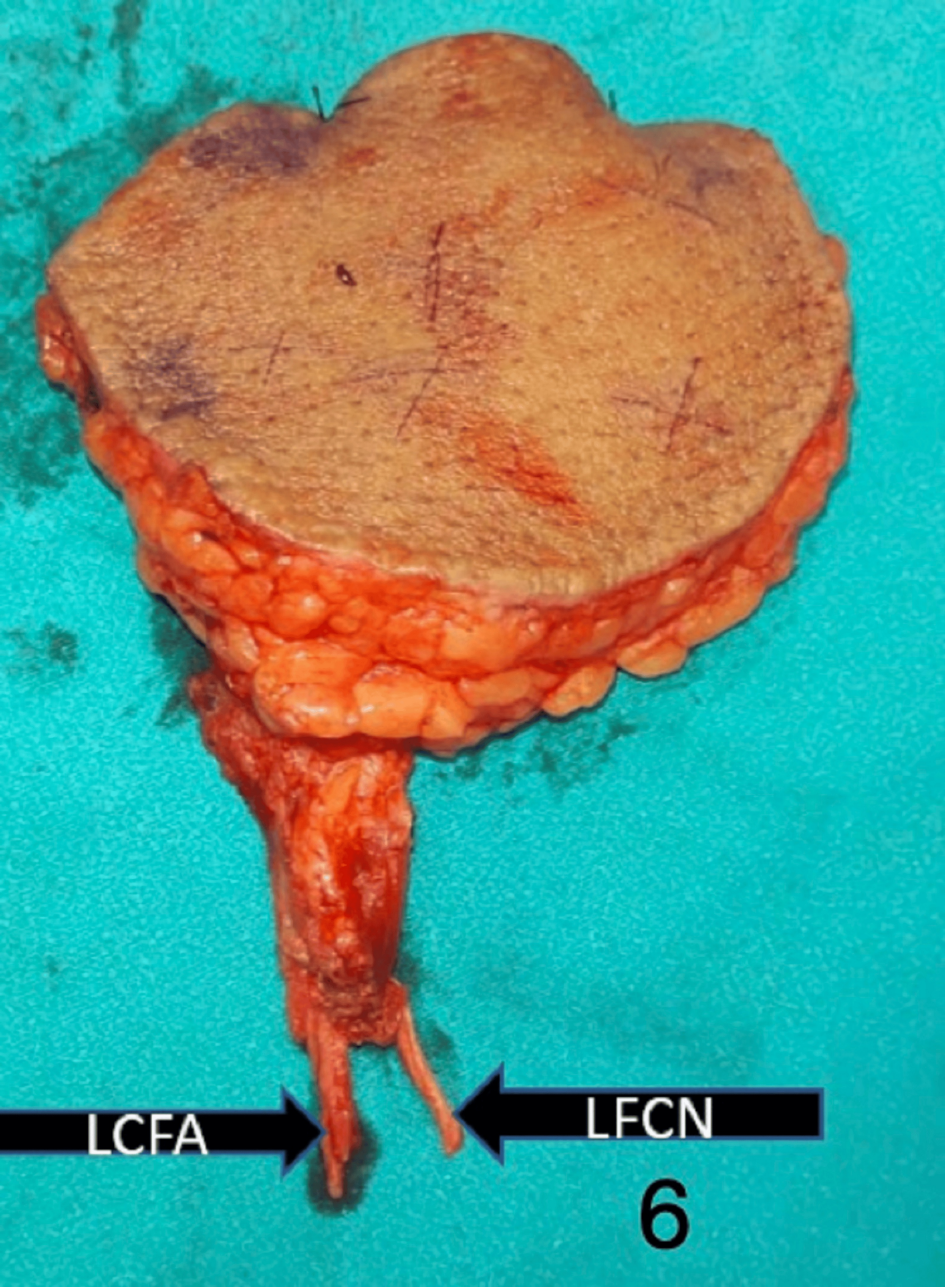
Intraoperative image showing the free ALT flap after harvest with vessels and the LFCN of the thigh ALT: anterolateral thigh; LCFA: lateral circumflex femoral artery; LFCN: lateral femoral cutaneous nerve

**Figure 7:**
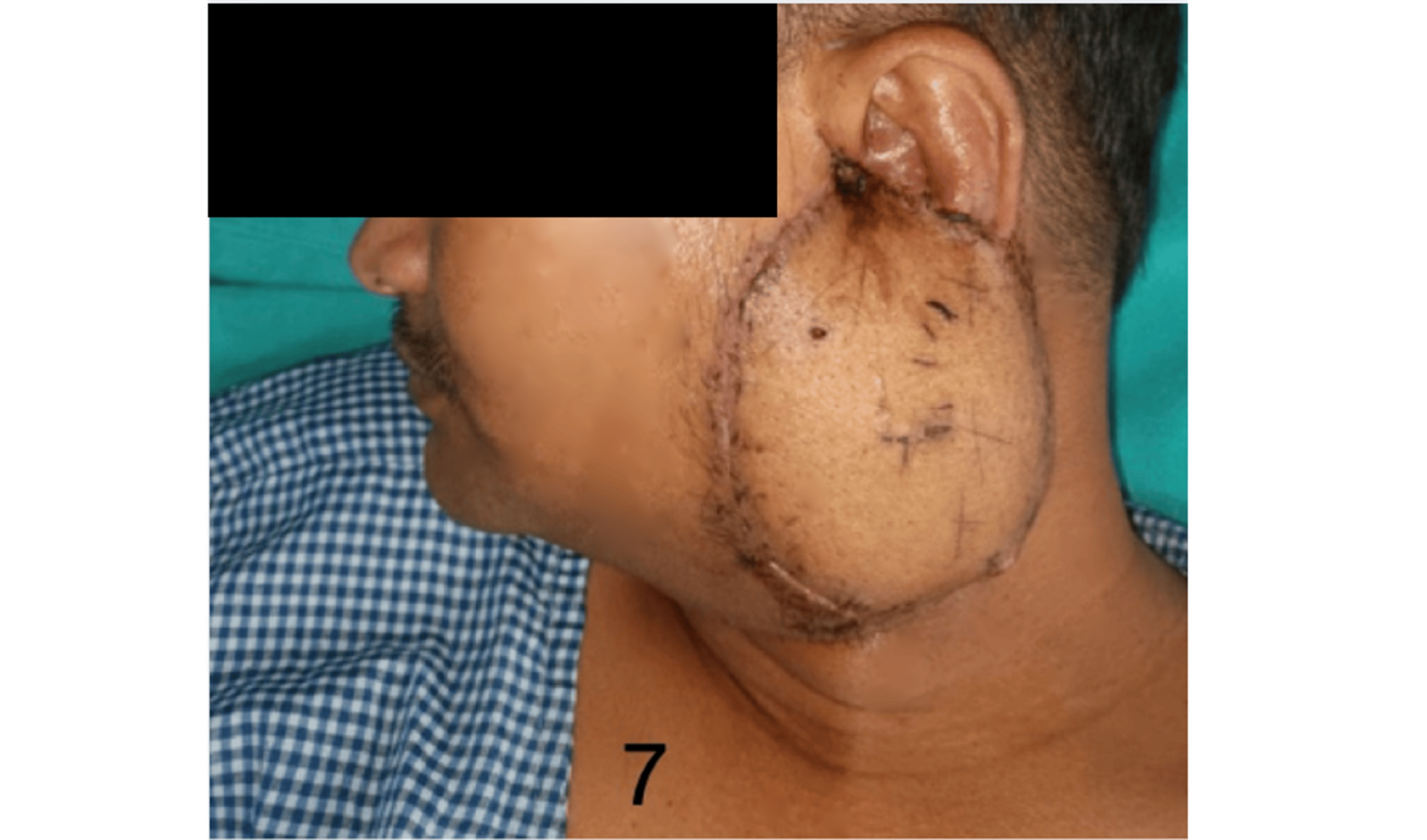
Follow-up image of the patient shown in Figure 5

Figures 5-7 show the preoperative, intraoperative, and postoperative images of a patient with BCC over the preauricular and retrooauricular area.

**Figure 8:**
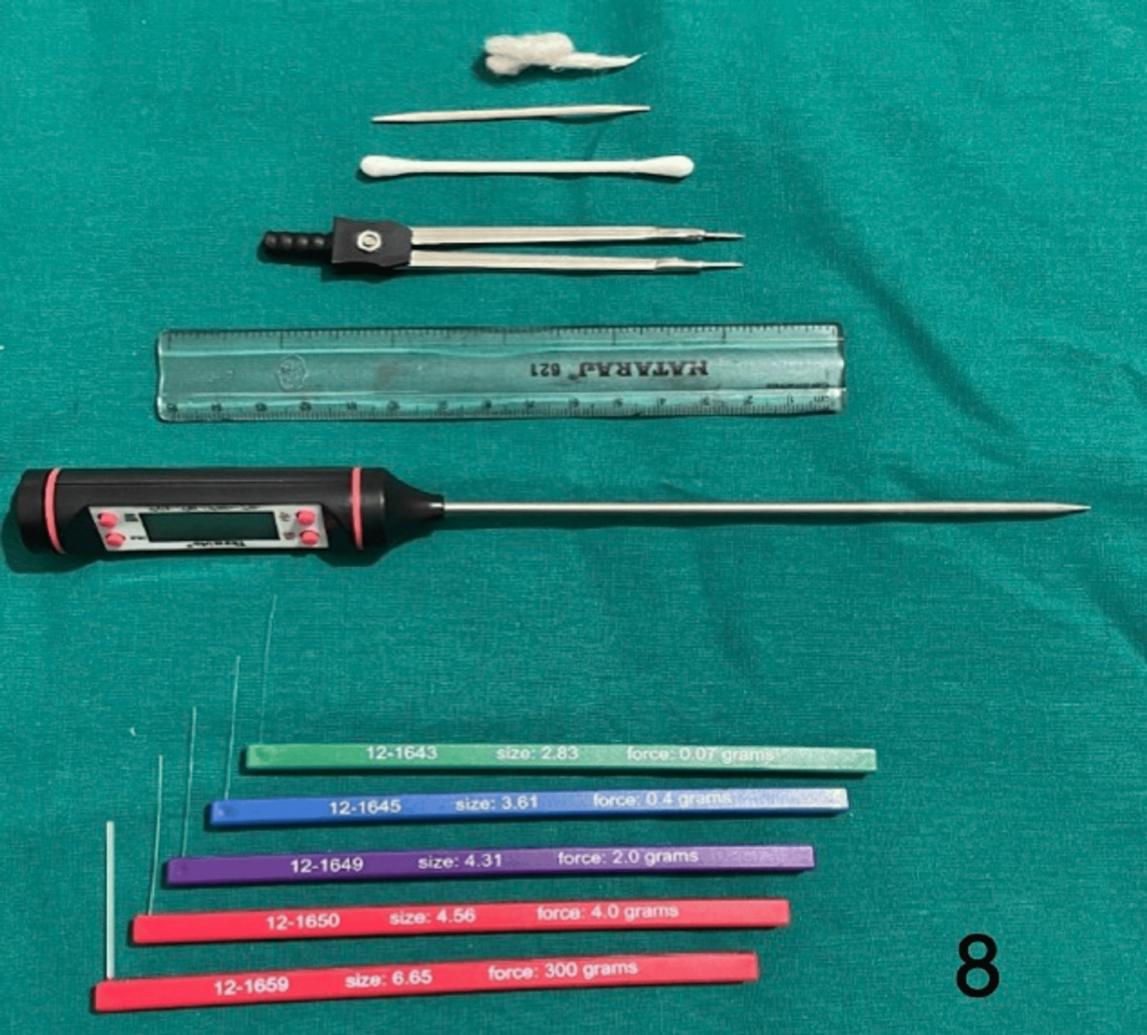
Instruments used for sensory assessment

Figure 8 shows the instruments that aid in a sensory assessment. 

**Figure 9:**
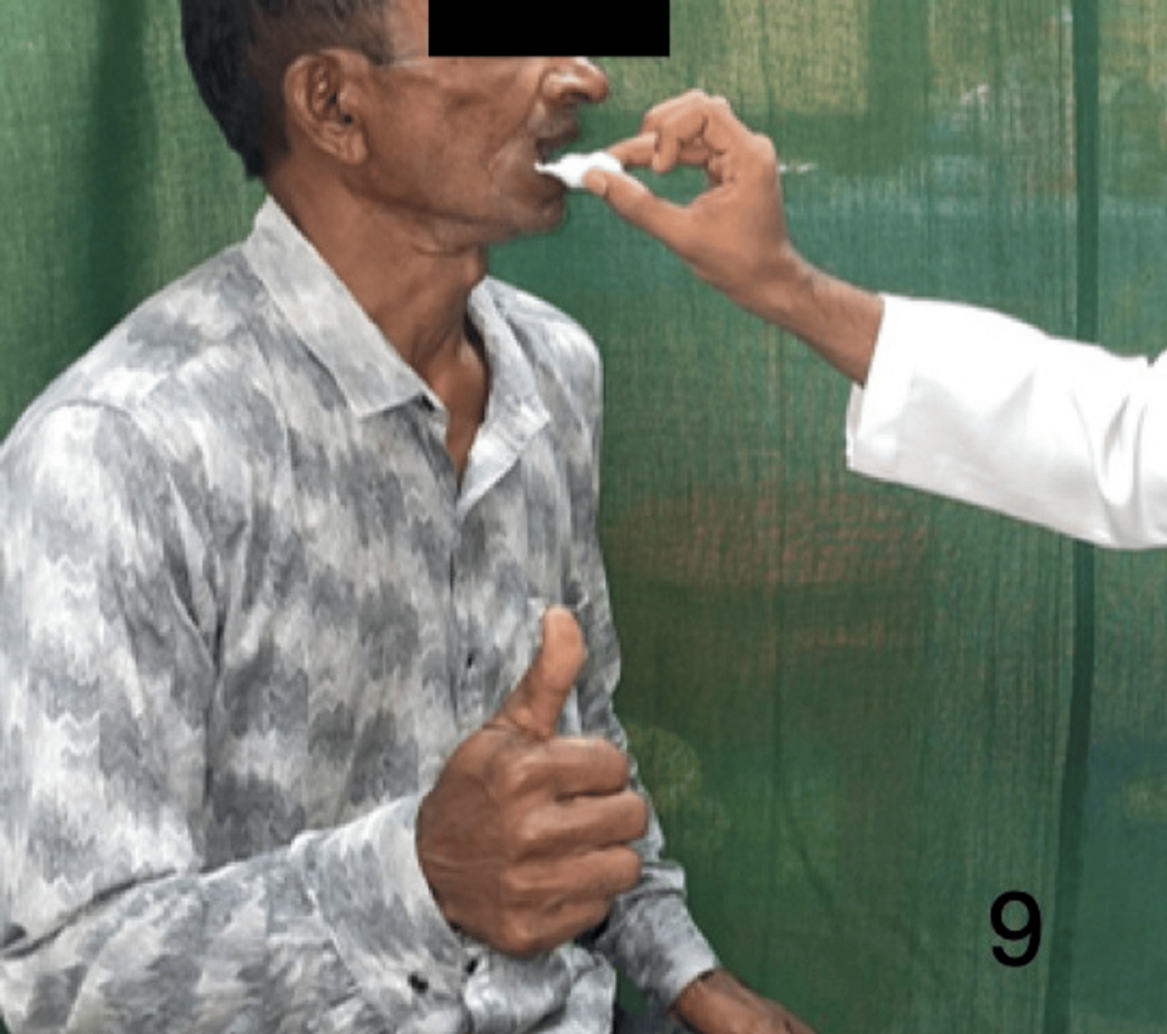
Demonstration of the superficial touch sensation over the flap

**Figure 10:**
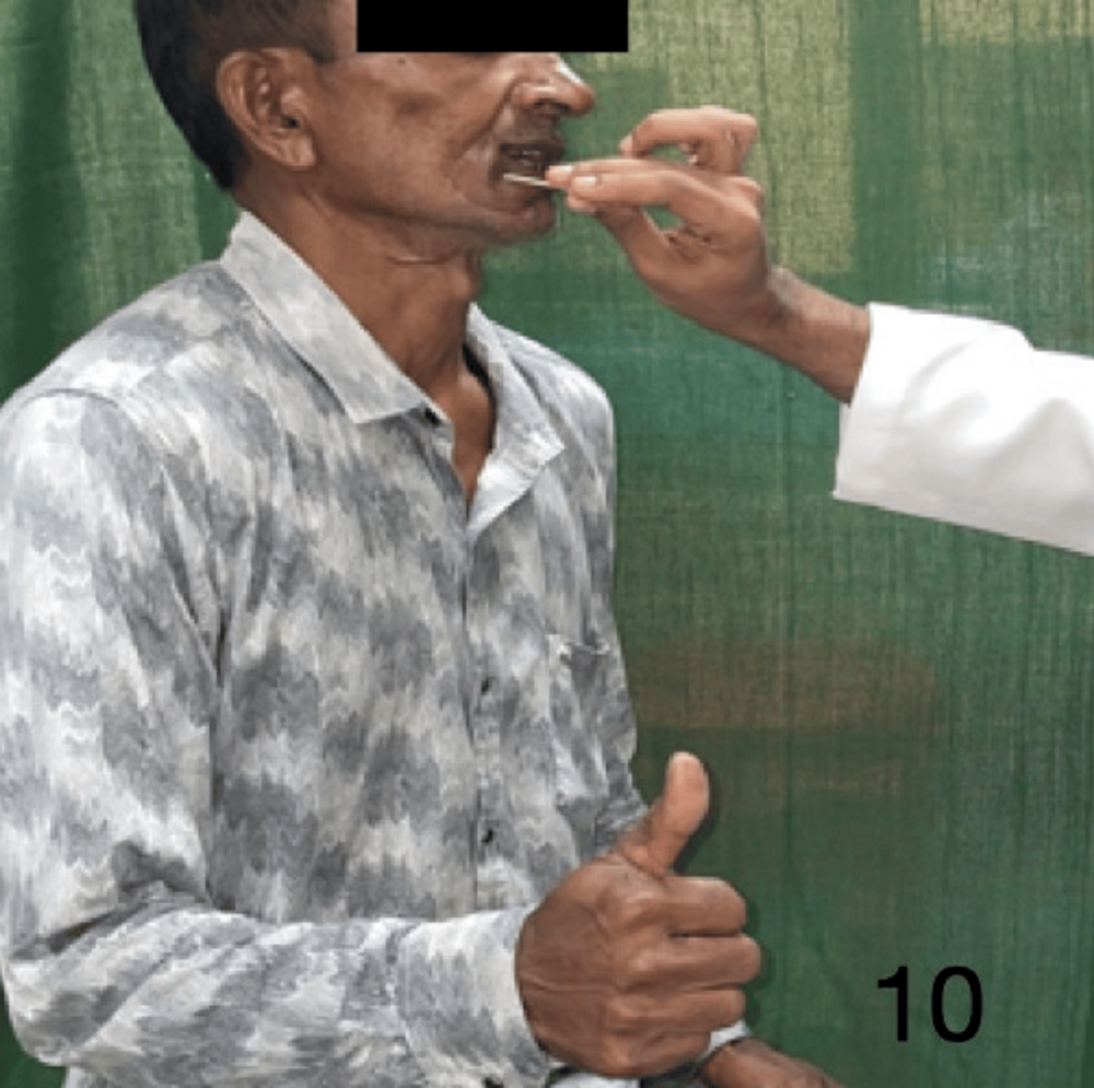
Demonstration of pain sensation over the flap

**Figure 11:**
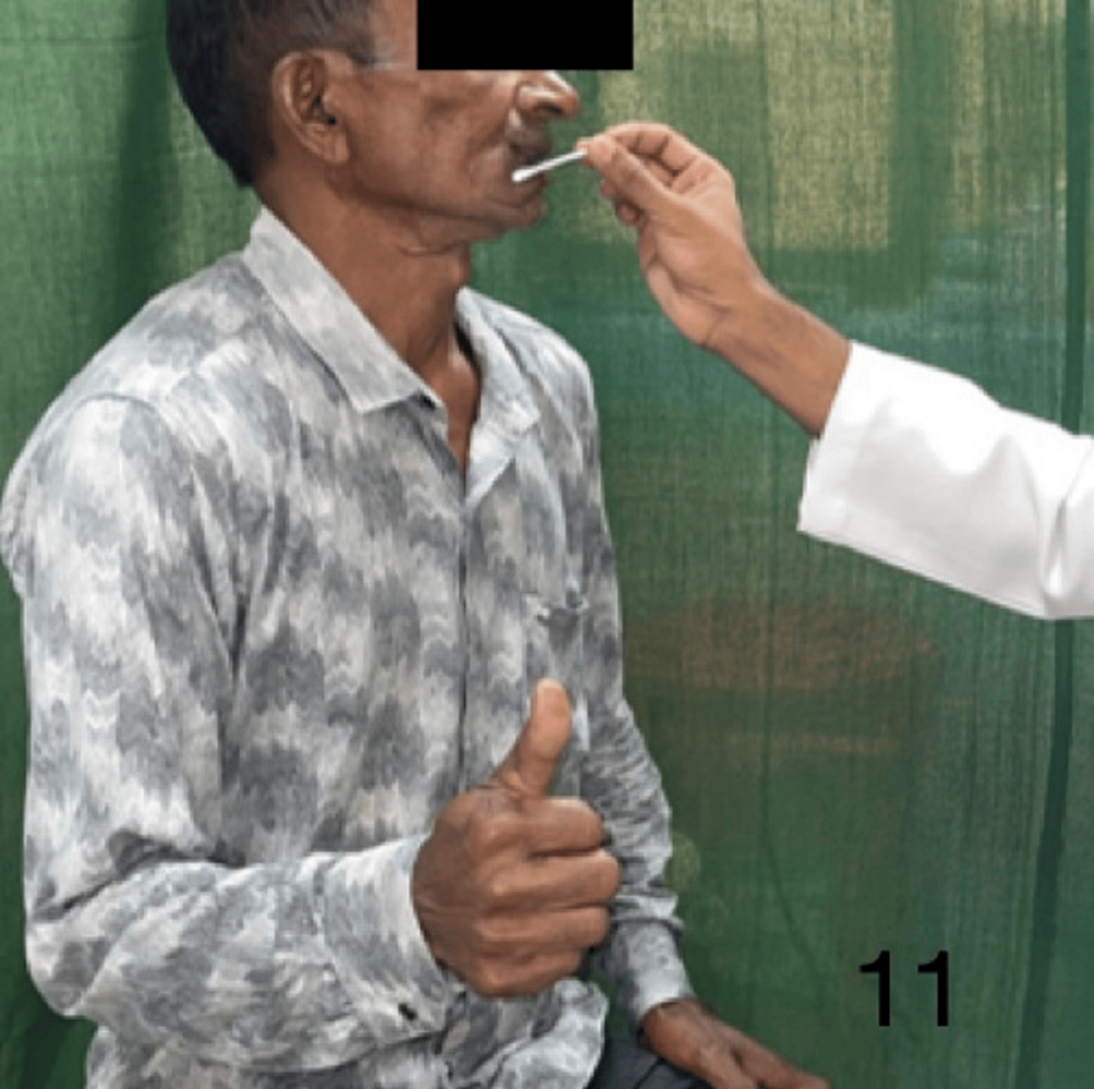
Demonstration of temperature sensation over the flap

**Figure 12:**
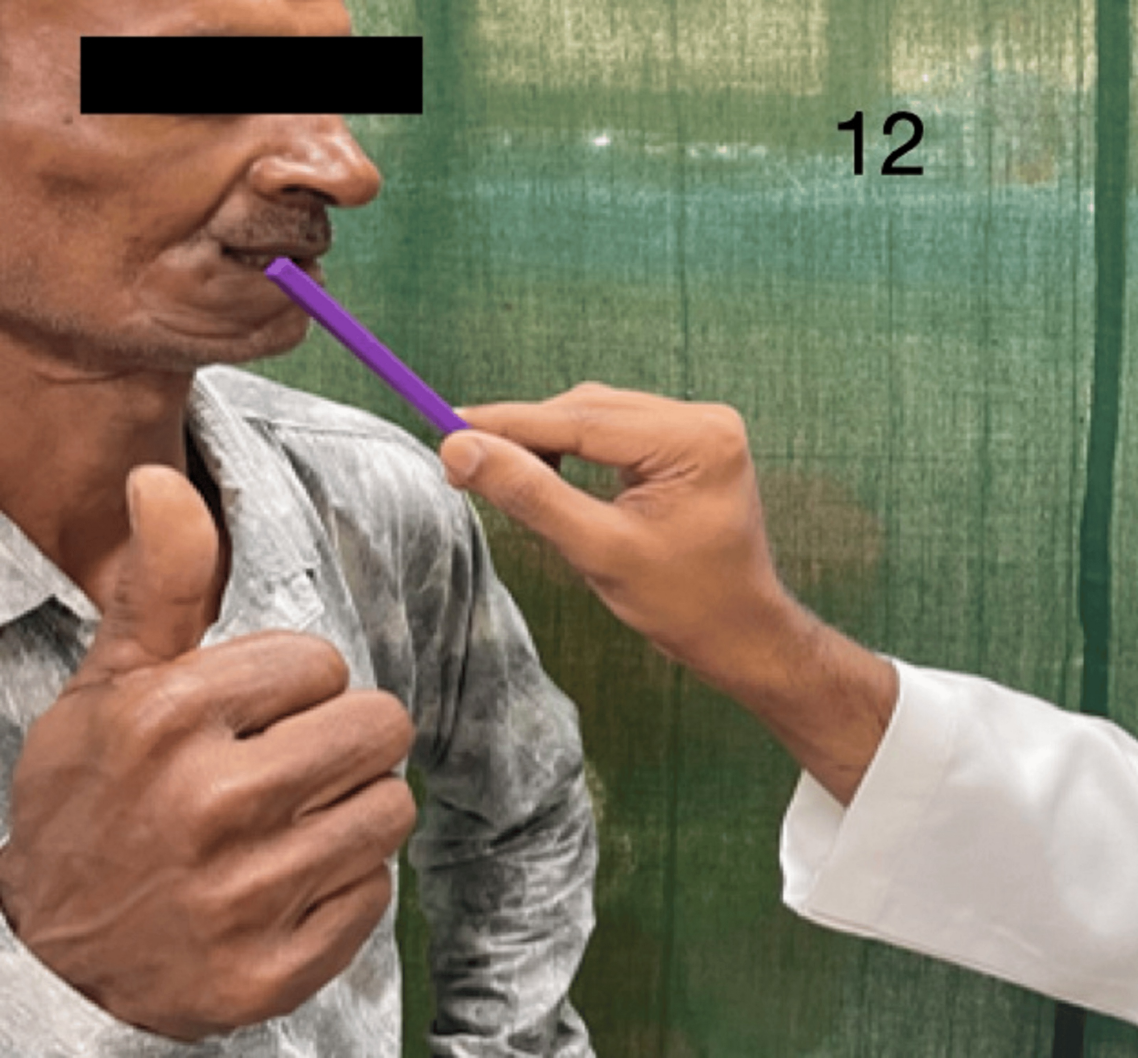
Demonstration of pressure sensation over the flap

**Figure 13:**
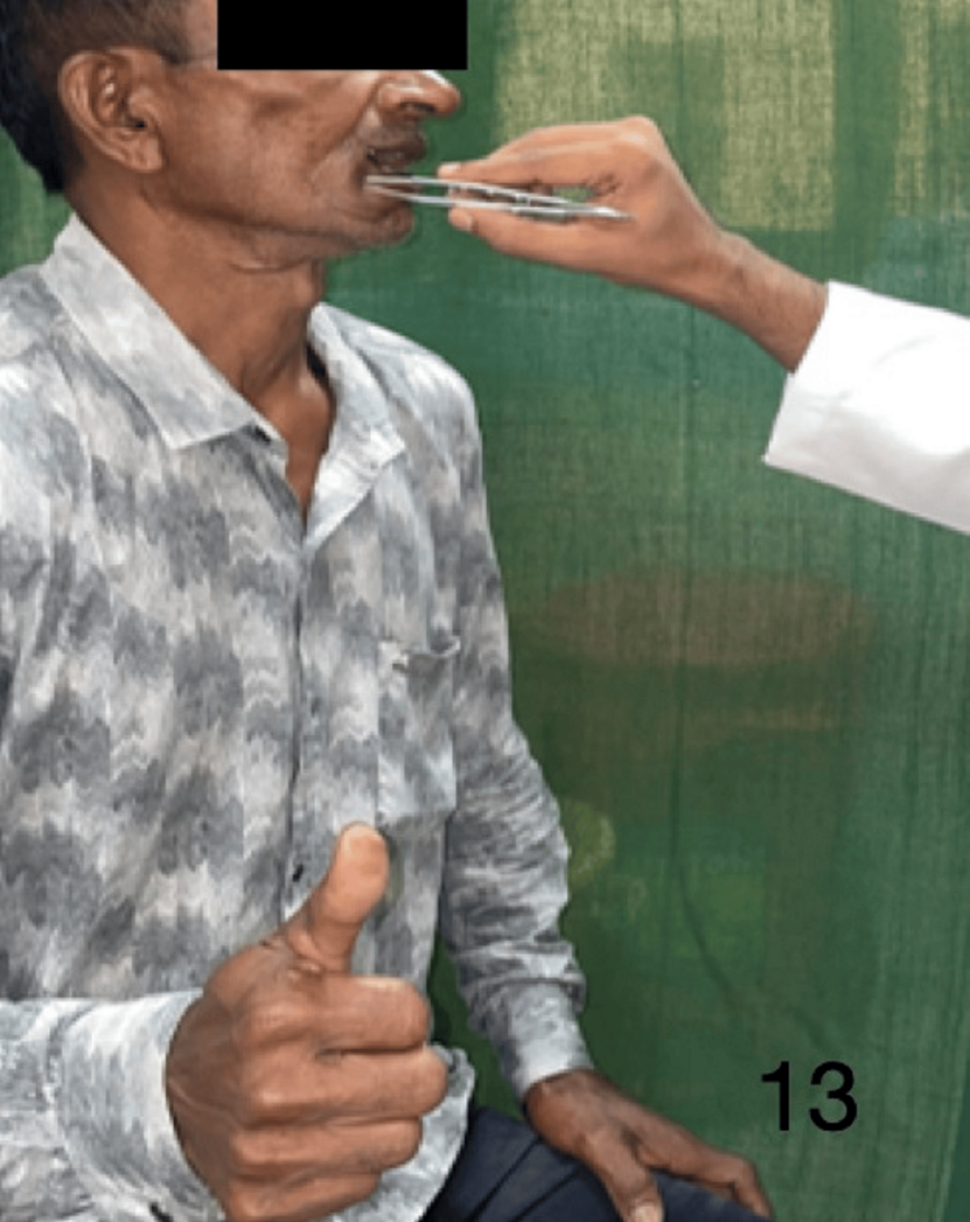
Demonstration of two-point discrimination sensation over the flap

Figures 9-13 demonstrate acknowledgment of the sensations of superficial touch, pain, temperature, pressure, and two-point discrimination.

## Discussion

The present study demonstrates a significant improvement in sensory recovery for all five modalities of sensation in free flaps at the six-month follow-up compared to the control group. This finding is consistent with the results reported in previous literature, which highlights the importance of sensory reinnervation in enhancing postoperative outcomes. Specifically, at six months, the study group showed full recovery of touch, pain, temperature, and pressure sensations, as well as 2-point discrimination abilities, significantly outperforming the control group. This marked improvement is evident from the statistically significant p-values obtained in all sensory modalities assessed, emphasizing the efficacy of sensory reinnervation in free flap reconstruction.

Namin AW et al. conducted an extensive literature analysis on functional and sensory recovery in neurotized and non-neurotized free flaps in the head and neck region [8]. In various works of literature, other nerves like the lingual nerve, branch of cervical plexus, posterior auricular nerve, inferior alveolar nerve, and hypoglossal nerve have been used as recipient nerves. Their findings revealed that sensate flaps exhibit superior static 2-PD and pressure sensations compared to insensate flaps. The current study further supports these findings by showing significant sensory recovery in the study population, suggesting that sensory reinnervation might hold an important role in enhancing sensory outcomes post-reconstruction.

In a study by Kuriakose MA et al., neurotized free flaps used for the reconstruction of tongue malignancy were evaluated for sensory recovery [9]. Their results indicated substantial sensory restoration within eight months, with sensory outcomes comparable to those of the normal tongue. The study suggested that the sensory recovery in innervated free flaps was almost equivalent to the native tissue. The sensory recovery in this study supports our study's findings of significant sensory improvements in the innervated free flaps. In the study by Kuriakose et al., pressure sensation was found to be 3.8 g in the flap area [9], in our study it was found to be 2.04g. This similarity suggests that reinnervation can effectively restore sensory functions in free flaps, contributing to better postoperative outcomes. A study by Rhee et al. emphasized the usefulness of sensory recovery in the upper aerodigestive tract. The electrophysiological mapping was done of neurosomes of potential sensate flap donor sites to ascertain their variations and hence aid in flap planning [10]. Baas M et al. conducted a comprehensive study comparing sensory reinnervation with no sensory reinnervation in several free flaps, including the RAFF, RA, ALT, and tensor fascia lata (TFL) flaps [11]. Their findings indicated superior sensitivity in reinnervated flaps, consistent with the present study's results. Moreover, their research suggested that sensory reinnervation might enhance overall tongue function and that factors such as sex, age, smoking, and postoperative radiation treatment did not significantly impact sensory recovery. These insights further validate the current study's outcomes, emphasizing the benefits of sensory reinnervation in free flap reconstructions. As most of the patients (93.33%) had undergone post-op radiotherapy, it has not halted the neurotization process.

Kim JH et al. compared the sensory recovery of sensate and non-sensate free flaps, finding significant differences in subjective sensory scores and 2-point discrimination (1.35 +/- 0.8 cm) capacities favoring sensate flaps [12]. This aligns closely with the present study, where the study group exhibited significantly better sensory recovery than the control group across most assessed modalities. However, in our study, the mean two-point discrimination was found to be 1.39 cm (static) and 0.72 cm (mobile). P values for both the 2-point discrimination were not found to be statistically significant (p- 0.75 for static and 0.125 for mobile). The presence of more noticeable nerve fibers in sensate flaps, due to innervation as reported by Kim JH et al., provides a possible explanation for the enhanced sensory outcomes observed in the present study, highlighting the importance of incorporating sensory nerves in free flap reconstruction.

Yu P's research on post-glossectomy reconstruction further supports the findings of the present study [13,14]. Yu P found that innervated flaps outperformed non-innervated flaps in all sensory recovery modalities, and postoperative radiation treatment might impede sensory recuperation. The current study's results, showing significant sensory recovery in the study group, align with Yu P's observations and suggest that sensory reinnervation contributes to improved functional outcomes and patient satisfaction. Overall, the present study's findings are consistent with existing literature, reinforcing the positive impact of sensory reinnervation on sensory recovery and functional outcomes in free flap reconstructions.

In another study by Boyd et al. where partial glossectomy and floor-of-mouth defects were reconstructed with free radial artery forearm flap. The lateral antebrachial cutaneous nerve was coapted to the lingual nerve and the results of sensory recovery were assessed. Results were found to be statistically significant as compared to non-innervated flaps [15].

In our study, we conducted the tests for all sensory parameters over GAN sensory territory in the study group over the ipsilateral flap area (where GAN fascicles were used as the recipient nerve) and compared it with the contralateral side. There was no clinical significance over GAN territory over the side where GAN fascicles were used as the recipient nerve as compared to contralateral normal GAN sensory territory. The mean pressure sensation in the periauricular area of the donor area was found to be 42.56 g and 2.85 g in the contralateral area, P value was found to be 0.178, which was not significant. Two-point discrimination values were found to be statistically not significant in both groups. However, when assessed with the Mackinnon Dellon Scale with S4 in all patients for the contralateral side and one patient with S3, 4 patients with S3+, and 10 patients with S4, significance was found (p-value 0.049), that is sensory recovery is inferior in the donor area as compared to the normal contralateral area. However, patients did not complain of much disturbing sensation and no patients complained of any discomfort in the donor area. In a study by Jeffrey et al., it has been assessed that nerve donor site complications are minor in all patients, which is found to be consistent with our study [16]. In a study by Brown et al., it was emphasized that the posterior branch of the greater auricular nerve if spared would cause very few sensory complaints in the periauricular area [17].

Reconstruction of the head and neck has extensively documented the restoration of sensation in noninnervated flaps. In non-innervated flaps, spontaneous reinnervation does happen, but it takes longer to develop, and it doesn't provide meaningful tactile sensation or two-point discrimination. It also doesn't have enough functional outcomes. The patient's capacity to manage oral secretions and food boluses depends on these sensory modalities.

The greater auricular nerve used in our study for neurotization of all the cases is a potential recipient nerve. This nerve is found in the vicinity, in the operative field, and two to three fascicles can be easily sacrificed without disturbing or distressing sensory complaints. There may be a few minimal complications like sensory loss in the GAN sensory territory and hyperesthesia due to neuroma formation, which can be observed in some patients [16].

There are very few studies on the greater auricular nerve as the recipient nerve. Many studies use the lingual nerve for sensory innervation. Our study gives an insight into the role of GAN as a potential recipient nerve. Also, the minimal sensory disturbance in the auricle suggests the salvageability of the fascicles in this nerve. Since in our study, sensory recovery in neurotized free flaps is similar to other previous studies using the lingual nerve, we can recommend GAN as a potential sensory donor nerve.

Limitations of the study

First, the sample size of the study and control groups may limit the generalizability of the findings. A larger sample size would provide more robust conclusions. Additionally, the follow-up period of six months, while adequate for assessing short-term outcomes, may not capture long-term changes in sensory recovery, warranting further long-term studies to evaluate sustained improvements.

## Conclusions

This study highlights the significant improvements in sensory recovery achieved through sensory reinnervation in free reconstructed flaps, emphasizing the importance of incorporating sensory nerves in reconstructive procedures for enhanced postoperative outcomes. Whenever possible, microvascular reconstruction can be made sensate, utilizing sensory nerves in the flap and anastomosing with a sensory nerve at the flap recipient site. GAN is a sensory nerve that is easily available at the recipient site and can be utilized for sensory recovery during free tissue transfer. As only a few fascicles of GAN are to be used for coaptation, sensory disturbance in the territory of GAN is not very disturbing to the patient. Further research with larger cohorts and longer follow-up durations is needed to strengthen the evidence base and guide clinical practice in optimizing sensory outcomes in free-flap reconstruction surgeries.
